# Psychosocial, clinical and demographic features related to worry in patients with melanoma

**DOI:** 10.1097/CMR.0000000000000266

**Published:** 2016-05-18

**Authors:** Zoe Rogers, Faye Elliott, Nadine A. Kasparian, D. Timothy Bishop, Jennifer H. Barrett, Julia Newton-Bishop

**Affiliations:** a Section of Epidemiology and Biostatistics, Leeds Institute of Cancer and Pathology, University of Leeds, Leeds, UK; b Discipline of Paediatrics, School of Women’s and Children’s Health, UNSW Medicine, The University of New South Wales, Sydney, New South Wales, Australia

**Keywords:** melanoma, worry, psychosocial, cancer, oncology

## Abstract

The aim of this study was to investigate clinical, demographic and psychosocial predictors of melanoma-related worry. A questionnaire-based study in a population-ascertained cohort of individuals diagnosed with melanoma in the previous 3–6 months was carried out to identify factors associated with worry about melanoma shortly after diagnosis. A total of 520 patients felt worried about their future with respect to melanoma and 1568 patients felt confident about their future with respect to melanoma. Worry was less likely in men with partners than women with partners [adjusted odds ratio (OR)=0.51, 95% confidence interval (CI) (0.39–0.67)], and increasing age was protective against worry [adjusted OR=0.96 per year, 95% CI (0.95–0.97)]. Worry was more likely for patients with stage III/IV melanoma [adjusted OR=1.90, 95% CI (1.41–2.56) compared with stages IB–IIC], melanoma arising in sun-protected sites (compared with a limb), no occupation (compared with workers), those who reported insufficient emotional support from healthcare providers [adjusted OR=2.20, 95% CI (1.56–3.09) compared with sufficient support], lower knowledge of melanoma [adjusted OR=4.50, 95% CI (2.82–7.18) compared with well informed], perceived financial hardship compared with no financial hardship and over three previous negative life events compared with none/one. Worry about melanoma outcomes after diagnosis is multifactorial in origin.

## Introduction

Cutaneous melanoma is the fifth most common cancer in the UK. Early detection and treatment has resulted in high survival rates, with 85% of patients surviving for at least 10 years 1. Despite this, a third of melanoma patients report clinically significant levels of psychological distress at diagnosis 2 and evidence suggests that they are seldom offered professional psychological support 3. Fear of recurrence and worry about how melanoma will impact on other areas of life are commonly reported as unmet needs 4. More information on the risk factors for melanoma-related worry is needed to help clinicians identify patients at risk of psychological distress 5,6.

An adapted version of the McCubbin and McCubbin resiliency model provides a theoretical explanation of the factors that predict fear of recurrence in cancer patients 7. This model proposes that personal factors (e.g. age, sex), current stressors (e.g. illness stressors, chronic stress) and lack of resources (e.g. financial, social support) contribute towards fear of recurrence 7. There is support for this in the melanoma literature: many studies have found that melanoma patients who are female and younger are more likely to report distress 8. Furthermore, healthcare-related stress has been found to be associated with distress in melanoma patients 9. Those who reported being provided sufficient information, and that their clinician was willing to discuss patient feelings, experienced lower levels of anxiety, although there was no adjustment for demographic nor clinical factors 9. Previous life stress in the form of negative events in the past 12 months was also a strong predictor of lower quality of life in melanoma patients 10.

More research is needed on the role of other potential stressors in adjustment to melanoma, including illness stressors, financial hardship, housing problems, job stress and parental responsibilities. The aim of this study is to investigate the relationship that these factors have with worry shortly after diagnosis in a population-based sample of melanoma survivors so that melanoma health care teams can better identify patients at risk.

## Methods

This large study investigated the risk factors for melanoma-related worry. We included demographic and clinical variables, perceptions of healthcare, previous life stress and known chronic stressors 11. Health locus of control measures were also included as a reported predictor of adjustment to cancer 12. We hypothesized that melanoma-related worry would be associated independently with advanced disease stage, female sex, having responsibility for children, youth, negative perceptions of healthcare, reported previous life stress and chronic financial stress.

### Study design

This was a questionnaire-based study in a population-ascertained cohort of individuals with melanoma, who were recruited to the Leeds Melanoma Cohort between 2000 and 2012, as reported previously 13. Patients aged 18–75 years, primarily resident in Northern UK and who had been diagnosed with cutaneous melanoma in the previous 3–6 months were eligible for the study. A total of 2184/3360 patients (65% participation rate) were recruited into the Leeds Melanoma Cohort study. Sex, age, site of primary, Townsend Index and Breslow thickness were investigated to ascertain whether there were differences between those who participated and those who declined to participate. Non-participators were significantly more deprived (more had scores at the upper end of the deprivation index – the Townsend Index) than participators. Non-participators also had significantly thicker tumours (measured by Breslow thickness) than participators. Sex, age, stage of disease and Townsend Index were investigated to ascertain whether they were determinants of response to the question on worry. Responders were significantly older (mean 55 years) and more affluent than nonresponders (mean 50 years).

Approval was obtained from MultiCentre Research Ethics (MREC/1/3/57) and the Patient Information Advisory Group [PIAG 3-09(d)/2003]. Participants were invited to complete validated and purpose-designed telephone-delivered and paper-based questionnaires (see Supplementary Information). Invitation to participate was issued 3 months after diagnosis.

### Materials

Data were collected on demographic factors, reported life events, perceived financial hardship, housing quality, job stress, multidimensional health locus of control 14, healthcare perceptions, melanoma-related knowledge, perceived social support (derived from the Norbeck questionnaire 15) and self-reported previous depression/anxiety (see Supplementary Information). Multidimensional health locus of control is a measure of the extent to which individuals believe that their health is or is not determined by their behaviour 14. The Norbeck Social Support Questionnaire is a multidimensional measure of positive social support (including affect, affirmation and aid) 15.

Melanoma-related worry was assessed using a single item; ‘How do you feel about the future with respect to the melanoma?’ Our use of a single item for detecting worry is supported by a meta-analysis that found ultrashort methods (including single-item questions) to be effective for detecting distress, depression and anxiety in cancer patients 16. Participants were defined as confident if they chose one of the following responses: ‘Very positive indeed’ or ‘Quite confident’. Participants were defined as being worried if they chose one of the following responses: ‘I don’t know’, ‘I feel quite worried’ or ‘I feel very worried’ (see Supplementary Information for more details of the variables in the questionnaire).

### Data analysis

Pairwise association between categorical variables was tested using Pearson *χ*^2^-tests and odds ratios (ORs), and 95% confidence intervals (CIs) were estimated from logistic regression models to assess the effects of predictor variables on the outcome measure of melanoma-related worry. More details are provided in the Supplementary Information.

## Results

Out of 2184 patients who participated, a response to the outcome question of melanoma-related worry was missing for 96 patients and thus these patients’ data were excluded from the analysis. Responses to the outcome question of melanoma-related worry were available for 2088 participants; 75% (*n*=1568) reported confidence about the future and 25% of participants (*n*=520) reported worry (Table 1). Responders were significantly older (mean 55 years) and more affluent than nonresponders (mean 50 years).

**Table 1:**
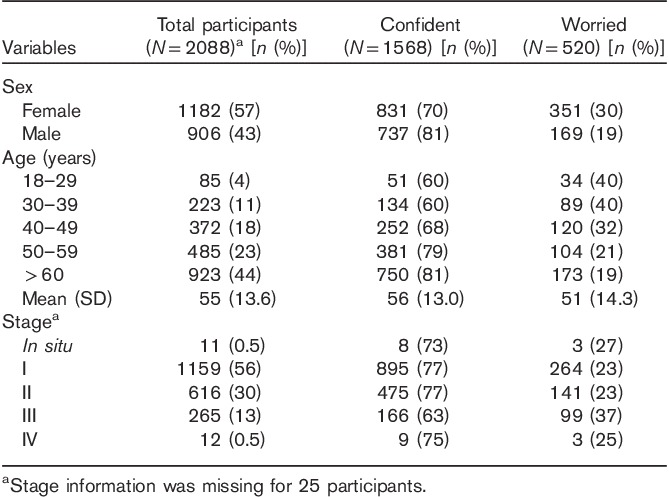
Demographic and clinical characteristics of the sample (*N*=2088); 75% of the sample (*N*=1568) reported confidence and 25% (*N*=520) reported worry

Men were less likely to report worry than women [unadjusted OR=0.54, 95% CI (0.44–0.67); adjusted OR=0.60, 95% CI (0.47–0.76), Table 2]. Men with a partner were less likely to be worried than women with a partner [unadjusted OR=0.47, 95% CI (0.37–0.60), *P*<0.001; adjusted OR=0.51, 95% CI (0.39–0.67), *P*<0.001; adjusted for age, stage, site of primary melanoma, length of hospital stay, employment status, Townsend Index and children under 18, results not shown in Table 2]. There was no difference in the likelihood of worry between single men and women [adjusted OR=1.06, 95% CI (0.62–1.81), *P*=0.84; test for interaction of the adjusted model, likelihood ratio test *P*=0.02, results not shown in Table 2].

**Table 2:**
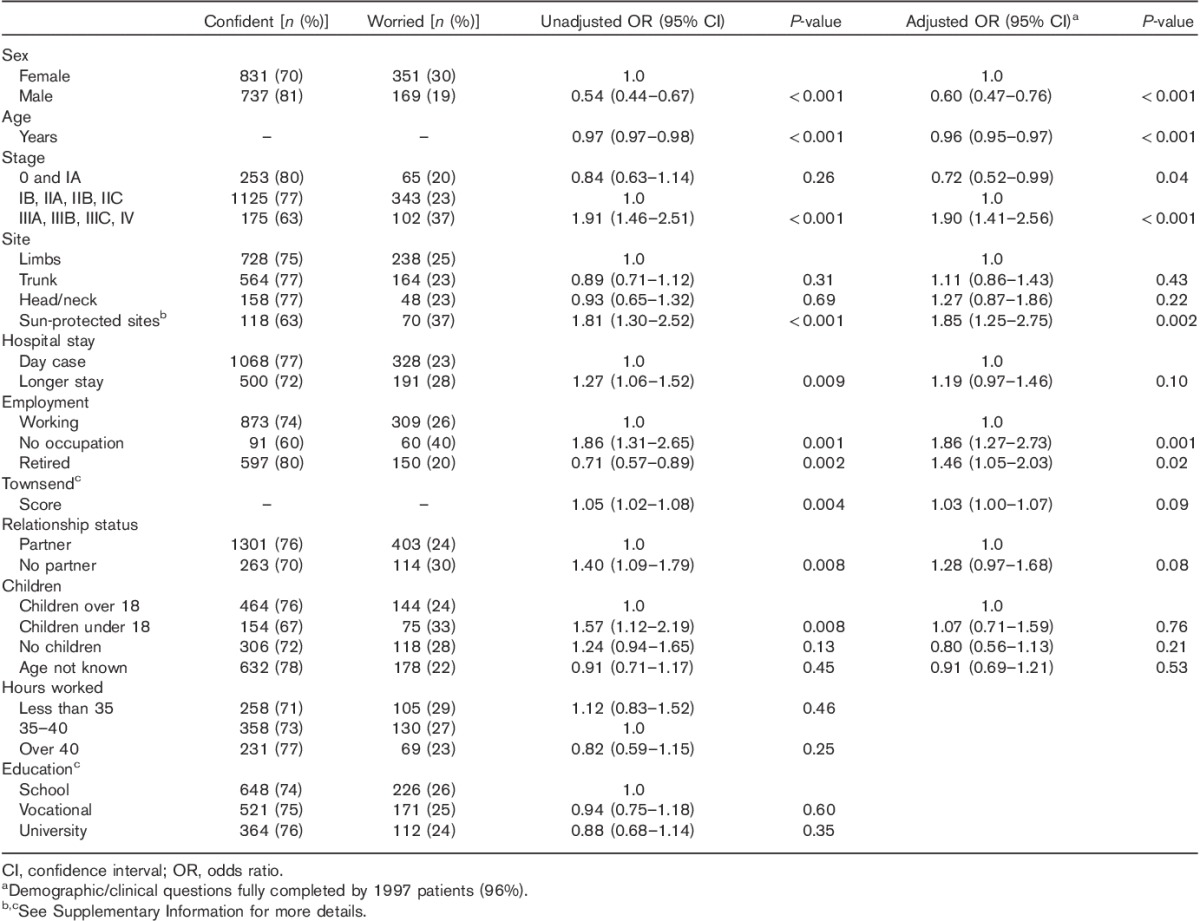
Odds ratios and 95% confidence intervals from univariable and multivariable analyses of the association between demographic/clinical factors and melanoma-related worry

The likelihood of worry decreased for each year increase in age [unadjusted OR=0.97, 95% CI (0.97–0.98); adjusted OR=0.96, 95% CI (0.95–0.97), Table 2]. Patients aged 18–45 years of age were more likely to be worried than patients older than 65 years of age [unadjusted OR=2.78, 95% CI (2.10–3.68); sex, stage and dependent children adjusted OR=2.94, 95% CI (2.10–4.12), result not shown in Table 2].

Patients with advanced stage disease (stage III/IV) were more likely to be worried than patients in the reference group of stages IB, IIA, IIB and IIC, and patients with melanomas in sun-protected sites were more likely to be worried than patients with melanomas on a limb (Table 2).

Of the 1182 patients with an occupation outside of the home, 1162 were employed and 20 were students. Patients who had no occupation outside the home were either unemployed (*n*=38), registered disabled (*n*=26) or home workers (*n*=87), and 747 patients were retired. Patients who had no occupation were more likely to be worried than patients with an occupation (Table 2). Only 17/574 patients (3%) who had no occupation or were retired and who responded to a question about job impact reported that their illness had prevented them from doing their job (result not shown in Table 2).

Retirement was protective for worry in unadjusted analysis, but was a risk factor for worry in multivariate analysis (Table 2). We investigated the relationship between retirement and worry in patients aged between 52 and 70 years as there were few patients older than 70 years of age who had an occupation and few patients younger than 52 years who were retired. Worry was more likely in patients who were retired compared with patients who were working within this age group [age-adjusted OR=1.92, 95% CI (1.25–2.93), result not shown in Table 2].

Factors that were associated with worry in unadjusted analysis, but not when included in multivariable analysis, included having children under 18 years of age, relationship status, length of hospital stay and Townsend Index (Table 2). Factors that were not associated with worry in unadjusted analysis were number of hours worked (for those with an occupation) and educational attainment (Table 2).

Patients who reported receiving no or poor support from their melanoma healthcare team were more likely to be worried than patients who were satisfied with the support provided [unadjusted OR=2.48, 95% CI (1.87–3.28); adjusted OR=2.20, 95% CI (1.56–3.09), Table 3]. In particular, patients who reported that they were told about their diagnosis unsympathetically were more likely to be worried [unadjusted OR=2.01, 95% CI (1.47–2.74); adjusted OR=1.71, 95% CI (1.17–2.49), Table 3].

**Table 3:**
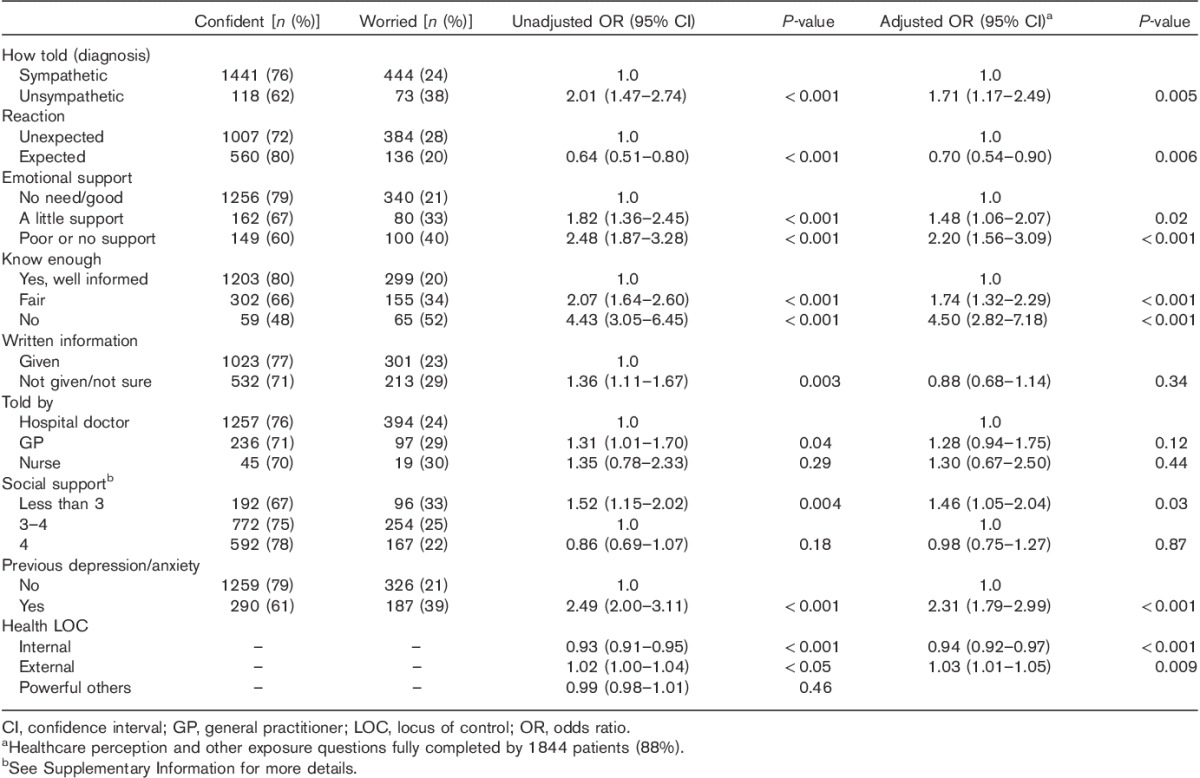
Odds ratios and 95% confidence intervals from univariable and multivariable analyses (age, sex and stage adjusted) of the association between healthcare perceptions, previous depression/anxiety, health LOC and melanoma-related worry

Patients who felt that they did not know enough about melanoma were more likely to be worried [unadjusted OR=4.43, 95% CI (3.05–6.45); adjusted OR=4.50, 95% CI (2.82–7.18)] than those who felt well informed (Table 3).

Factors that were associated with worry in unadjusted analysis but not when included in multivariate analysis were lack of written information and being told the diagnosis by the general practitioner compared with a hospital doctor (Table 3). Social support, previous depression/anxiety and health locus of control were included in the multivariable analysis to adjust for their association with worry (Table 3).

Patients were more likely to be worried if they reported financial hardship [unadjusted OR=2.05, 95% CI (1.55–2.70); adjusted OR=1.42, 95% CI (1.02–1.98), Table 4] and more likely to report current financial hardship if their illness had slight (*n*=76, 40%) or moderate/severe impact (*n*=49, 64%) on their finances (variable not shown in Table 4) than if they reported little or no financial impact (*n*=146, 11%), *χ*^2^(4)=244.6, *P*<0.001 (result not shown in Table 4).

**Table 4:**
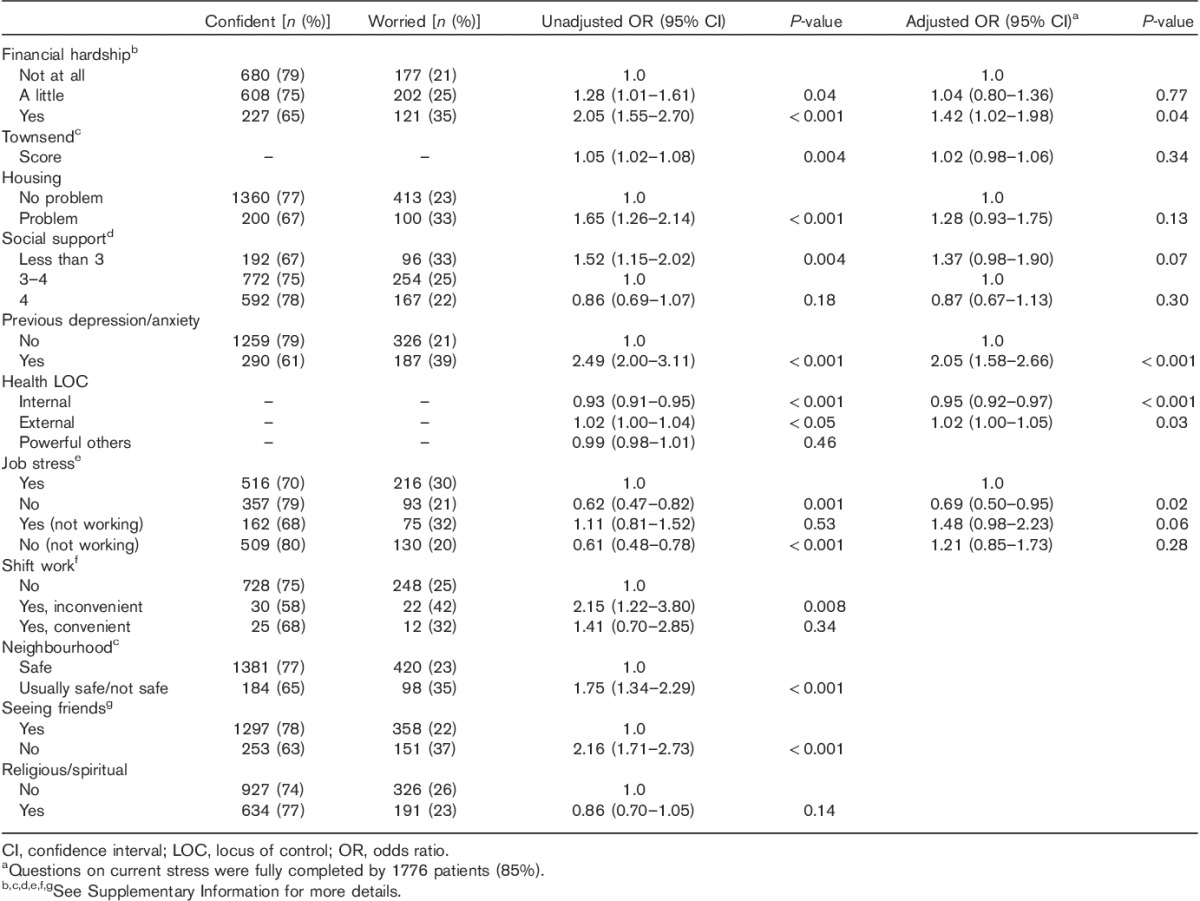
Odds ratios and 95% confidence intervals from univariable and multivariable analyses (age, sex and stage adjusted) of the association between financial hardship, deprivation, job stress and social support on melanoma worry

Some nonworkers (including retirees) reported work-related stress and were more likely to report melanoma-related worry (see variable job stress in Table 4). We investigated the relationship between job stress and worry in patients aged 45–65 years. In this group, nonstressed workers were less likely to be worried than stressed workers [age adjusted OR=0.54, 95% CI (0.35–0.82), result not shown in Table 4]. Stressed nonworkers were more likely to be worried than stressed workers [age adjusted OR=2.12, 95% CI (1.28–3.50), result not shown in Table 4].

Patients who had experienced previous depression/anxiety were more likely to be worried [unadjusted OR=2.49, 95% CI (2.00–3.11); adjusted OR=2.05, 95% CI (1.58–2.66)] than those who had not experienced previous depression/anxiety (Table 4). Internal health locus of control was protective against worry, whereas external health locus of control increased the likelihood of worry (Table 4).

Factors that were associated with worry in unadjusted analysis but not when included in multivariate analysis were Townsend Index, housing problems and having a smaller social network (Table 4). Shift work that was reported as inconvenient was associated with melanoma-related worry in unadjusted analysis (Table 4), but was not significant in an analysis adjusting for age, sex, stage, financial hardship and Townsend Index (result not shown in Table 4). This variable was not included in the multivariable model because of missing data. Reports of being unable to see friends and lack of neighbourhood safety were also associated with worry, but were not included in the multivariable analysis because of collinearity. Having religious/spiritual beliefs was not associated with worry in unadjusted analysis (Table 4).

Patients were more likely to report worry if they had experienced over three negative life events in the last 5 years compared with none/one negative life event [unadjusted OR=2.56, 95% CI (1.80–3.64); adjusted OR=1.67, 95% CI (1.10–2.55), result not shown in Table 4]. There was no significant difference in the likelihood of worry between patients who had experienced 2–3 negative life events and those who had experienced none/one life event.

The univariable and multivariable analyses are reported in Tables 2–4 and show that there were many independent factors predicting melanoma-related worry.

## Discussion

To our knowledge, this is the largest study of its kind to identify a broad range of psychosocial factors associated with worry in individuals with melanoma at diagnosis. The sample was felt to be reasonably representative of individuals with melanoma in the UK, being largely population ascertained, with a high participation rate and a mean age of 55 years at diagnosis (Table 1).

Limitations of the study were identified: the lack of validated questionnaires available to measure lifestyle-related stress and melanoma-related worry at the time of study conception meant that exposure questions were purpose-designed and piloted in a relatively small number of patients. Subjective patient perceptions of the quality of healthcare communications were utilized as we could not assess this objectively. Financial hardship was based on reports of the existence of debt or redundancy, but also utilized subjective perceptions of their severity and ability to manage on income. Finally, we could not corroborate reports of previous depression/anxiety with objective records of the patient’s psychiatric history.

Men with partners were less likely to be worried than women with partners, with no difference in worry between single men and women (Table 2). Married men report more health monitoring from their spouses than married women 17, and more spousal support has been reported by male colorectal cancer patients 18. We hypothesize that men with melanoma may benefit from more perceived spousal support than do women, and observe that those without partners are also at risk of worry.

Younger patients were more likely to be worried than older patients as reported previously 19, and this was independent of dependent children. Younger age is reliably associated with greater distress among cancer patients 20,21 and has been attributed to greater fear of recurrence, fear of death 22,23 and concerns about reproductive, lifestyle and career limitations 24. We suggest that younger individuals with melanoma tend to experience greater worry because of similar concerns.

Nonworkers and retirees were more likely to be worried than workers/students with adjustment for age (few nonworkers reported that melanoma had affected their ability to work). Unemployment is associated with reduced psychological well-being in healthy populations 25, as well as in a previous study of melanoma patients 26 and in the present study. Retirement is believed to be associated with psychological distress in both men 27 and women 28. To our knowledge, this is the first study to identify nonworkers and retirees as vulnerable to melanoma-related worry, independent of demographic and disease factors. We speculate that work distracts from worry and the support of colleagues may be a coping resource for patients in work.

Patients with advanced-stage melanoma (stage III/IV) were more likely to report feeling worried than those with stages IB–IIC. Although this finding is inconsistent with studies that found no association between melanoma stage and distress 2,19, the finding is intuitive and may reflect the considerable size of the study.

Melanoma in a sun-protected site was a risk factor for worry compared with melanoma on a limb. There is difficulty in monitoring melanomas in sun-protected sites (e.g. in the vagina, mouth or sinuses), and their rarity means that specific written patient information is not as readily available. Surgery may be disfiguring, is less likely to be curative and prognostic estimation is less certain than for common types of melanoma. To our knowledge, this is the first study to identify that patients with melanoma in sun-protected sites, understandably, express higher levels of worry. We therefore recommend that clinicians ensure that these patients are provided as much verbal and written information as possible about their particular types of melanoma, as well as explaining how to manage difficulties with self-examination in follow-up.

Patients who reported being told the diagnosis unsympathetically, not knowing enough about melanoma and a lack of emotional support from healthcare providers were more likely to report worry compared with those who reported more positive appraisals (Table 3). Evidence suggests that patient-centered communication is preferable to emotion-focused or disease-focused communication styles when breaking bad news 29. Satisfaction with information is also associated with lower anxiety 9,30. Although we cannot exclude the possibility that some patients inclined to worry may be more likely to experience communication as poor, these findings support an argument for healthcare teams to manage worry at the time of diagnosis by using appropriate communication styles and adequate information-giving. It is suggested that clinicians can alleviate the extent to which melanoma patients perceive their illness as threatening by preparing them in advance for a potential diagnosis if this seems likely, communicating diagnoses using a patient-centred style, providing emotional support if needed and ensuring that patients are well informed about their disease.

Patients who reported financial hardship were more likely to report worry than patients with no financial hardship with adjustment for demographic and clinical factors (Table 4). They were also more likely to report that their illness had an impact on their finances. Having cancer can introduce a significant financial burden for patients who may be unable to maintain their income (e.g. lack of sickness cover) and meet extra expenses 31. This suggests that patients with financial hardship are a particularly vulnerable group.

Patients who reported more than three previous negative life events were more likely to be worried than those reporting none/one life event, as has been reported previously 10. Patients with more than three previous life events were also more likely to report current financial hardship in the present study. These findings support the argument that non-cancer-related stress may precipitate a greater or more complicated stress response to a diagnosis of melanoma.

It is recommended that when screening melanoma patients for distress, previous stressful life events and current chronic sources of stress such as financial hardship, work-related stress, unemployment and lack of occupation more generally are taken into account, as well as the more established factors such as age, sex, relationship status and disease stage. Melanoma patients who present with these risk factors may benefit from help to manage current additional sources of stress within the context of melanoma treatment (e.g. help with travel/medication costs, letters to employers for time off work). Furthermore, individual attitudes to health may indicate vulnerability to worry and patients who have high external health locus of control may benefit from education on maintaining healthy lifestyles and the importance of self-examination. Finally, those patients with previous experience of depression and anxiety may benefit from increased access to psychological support services.

This population-based study has highlighted numerous clinical, demographic and psychosocial risk factors, from various life domains, that were strongly associated with worry in this large sample of individuals with melanoma, potentially allowing for the identification of vulnerable patients in clinic. A weakness of the study is the subjective and multifactorial nature of some of the variables and their complex interrelationships. Despite this, the study supports the view that individuals with melanoma who worry most about the future may do so because of the cumulative effects of stress (both disease and noncancer related) and a perceived lack of support from healthcare teams and within their lives generally. Our analyses show that these risk factors are independent, and the findings are consistent with experience in clinic where those patients who appear to cope less well are those for whom many stressful factors have contributed. The study supports the McCubbin and McCubbin resiliency model of the factors that predict fear of recurrence in cancer patients 7. That previous anxiety/depression and a high external health locus of control were also risk factors suggests that individuals differ (as expected) in their ability to cope.

## Supplementary Material

All supplementary digital content is available directly from the corresponding author.
